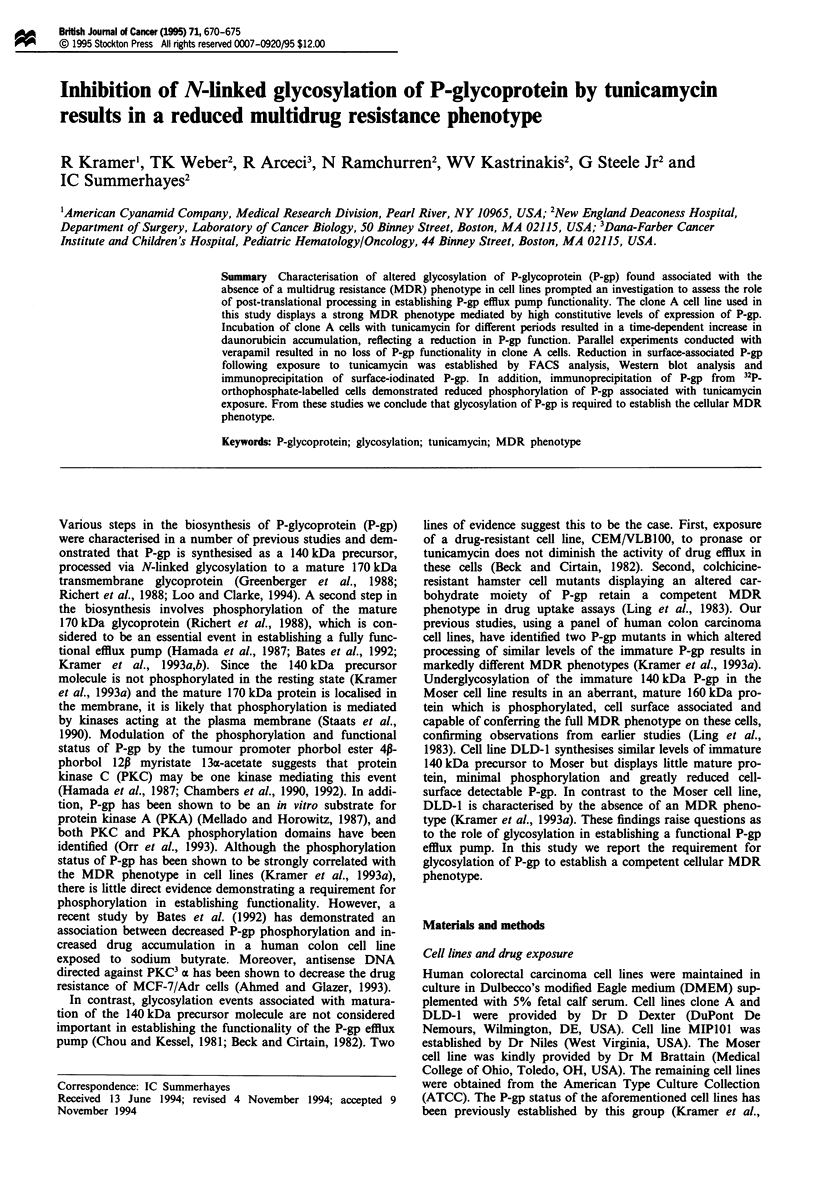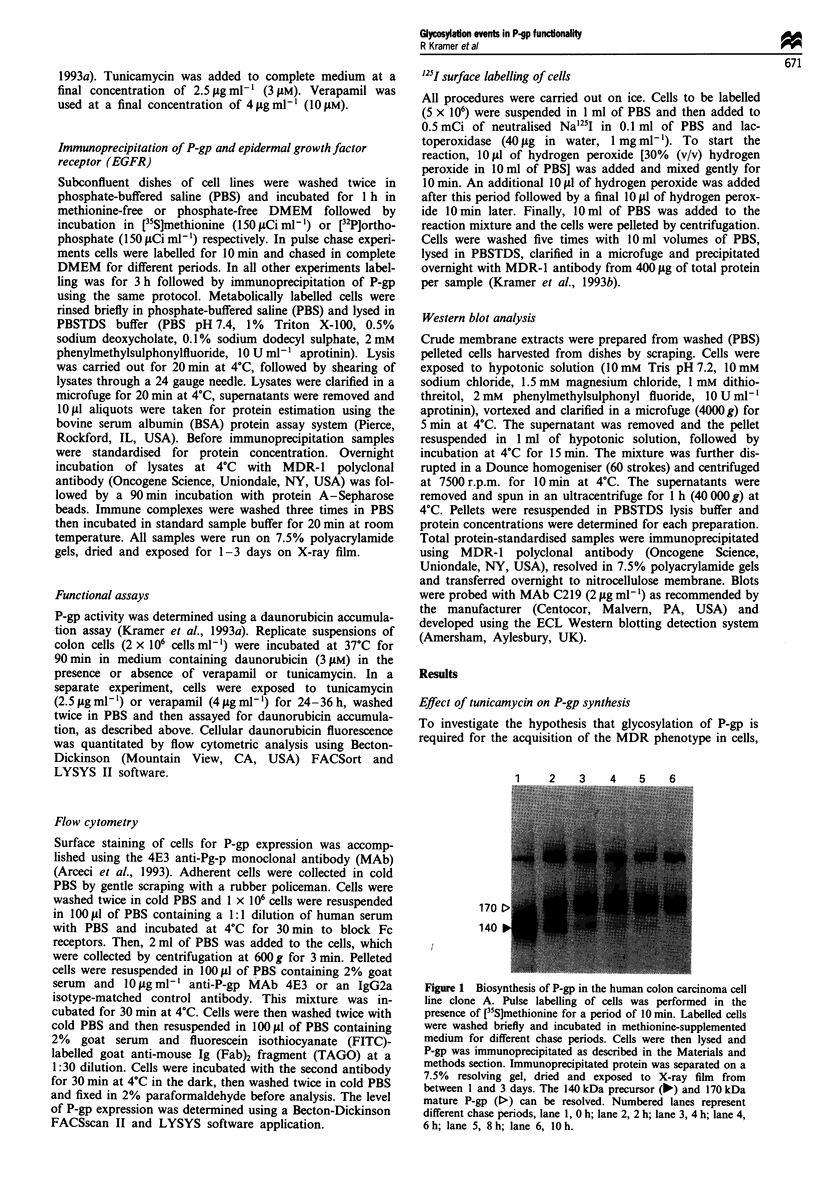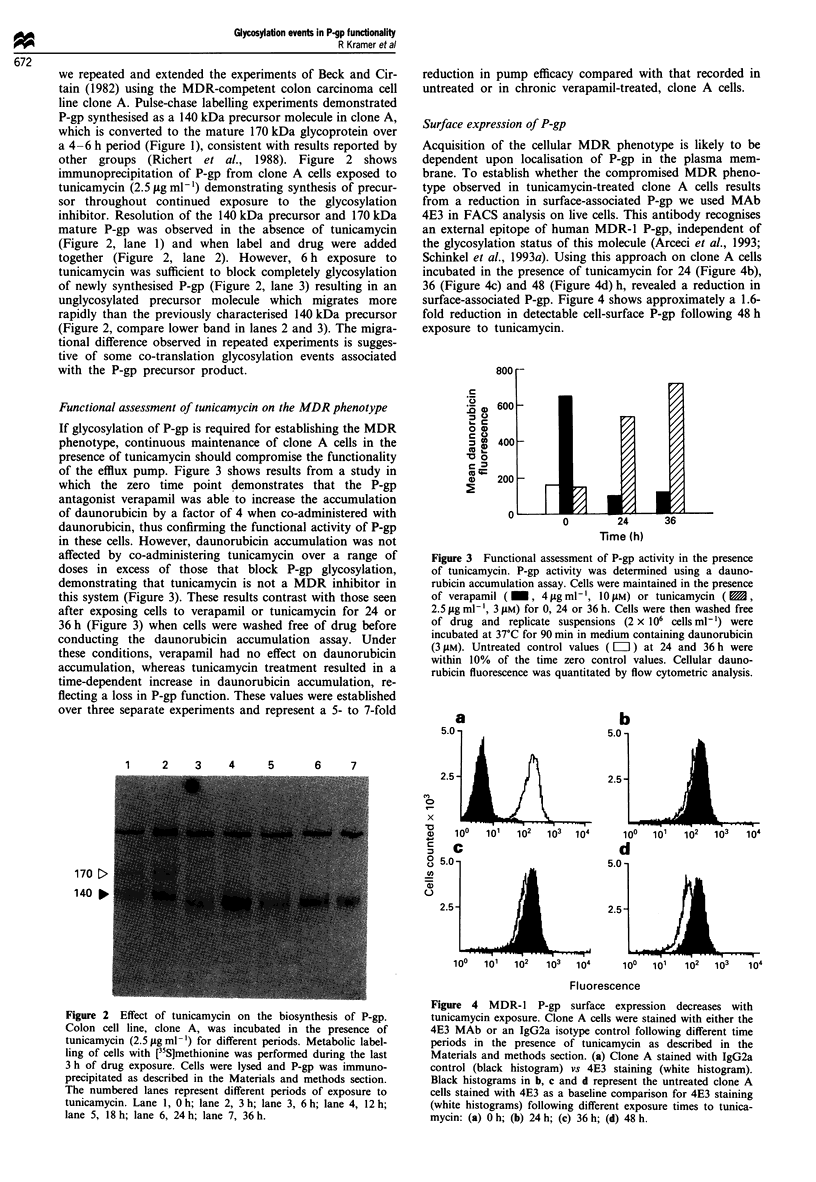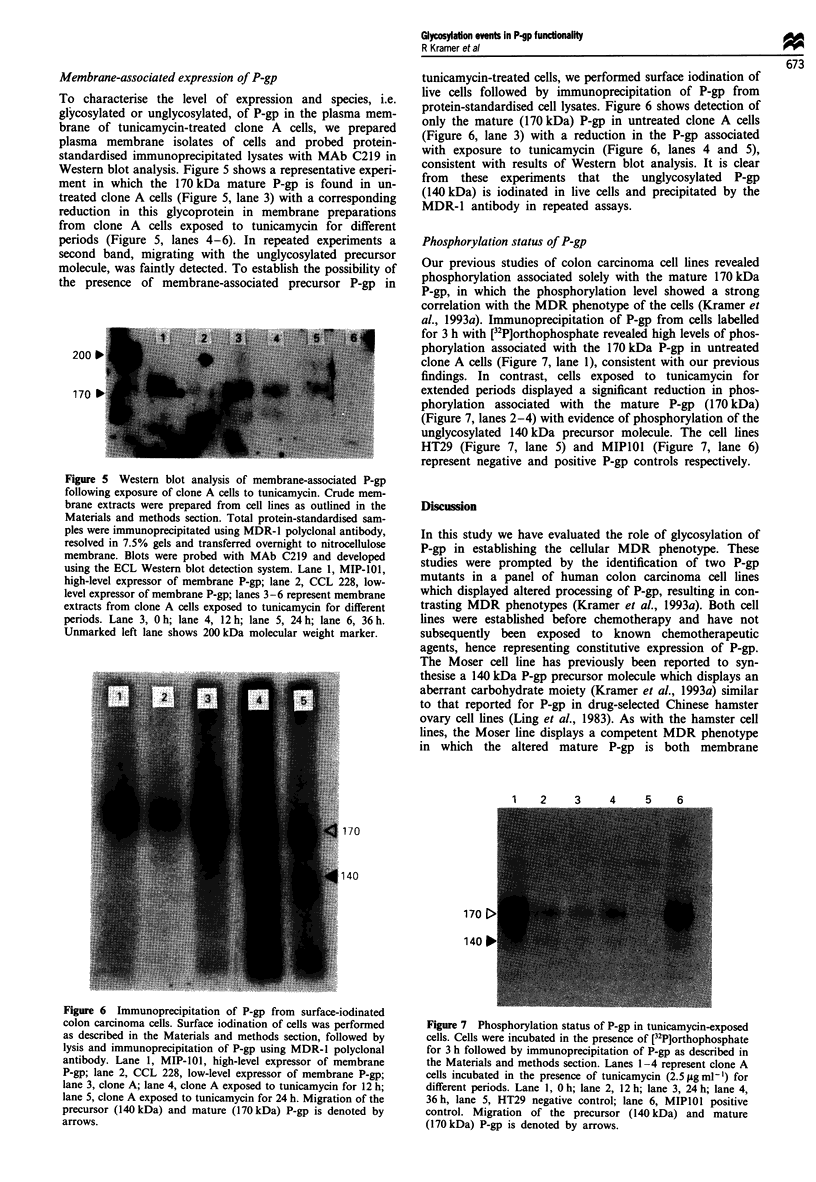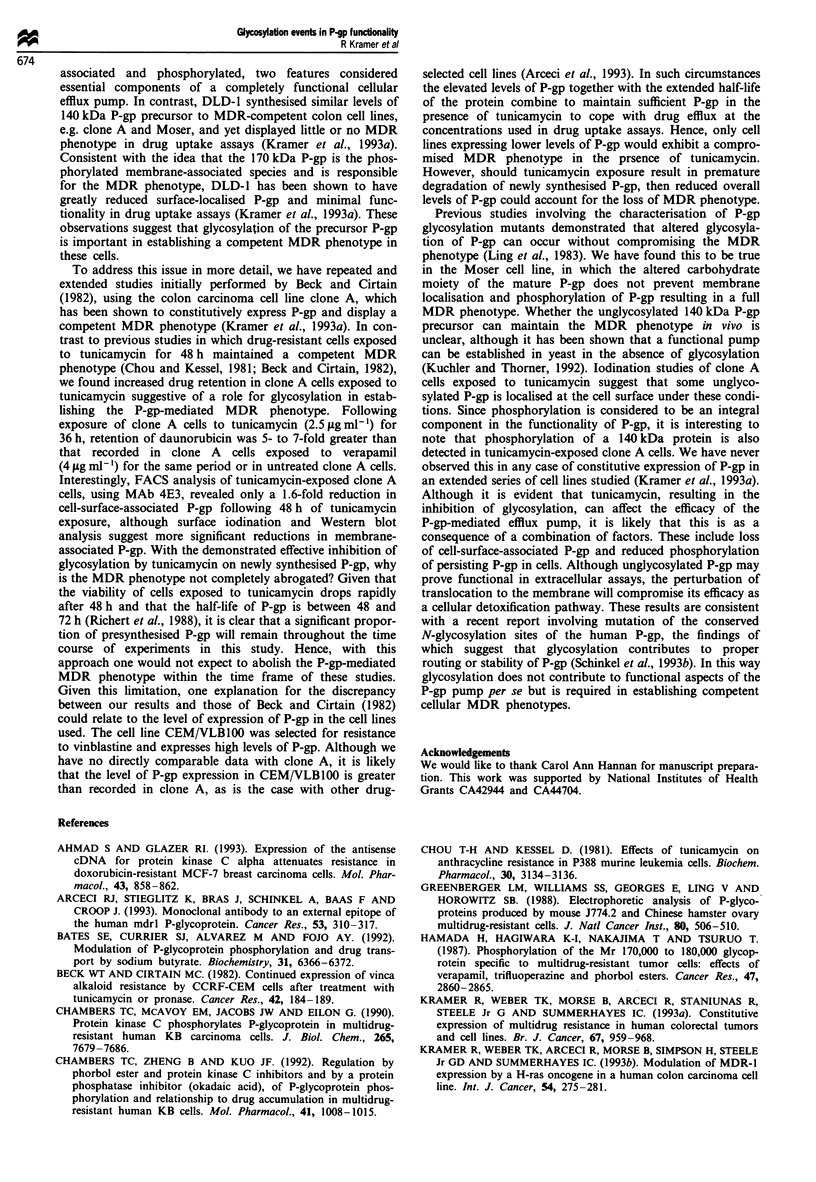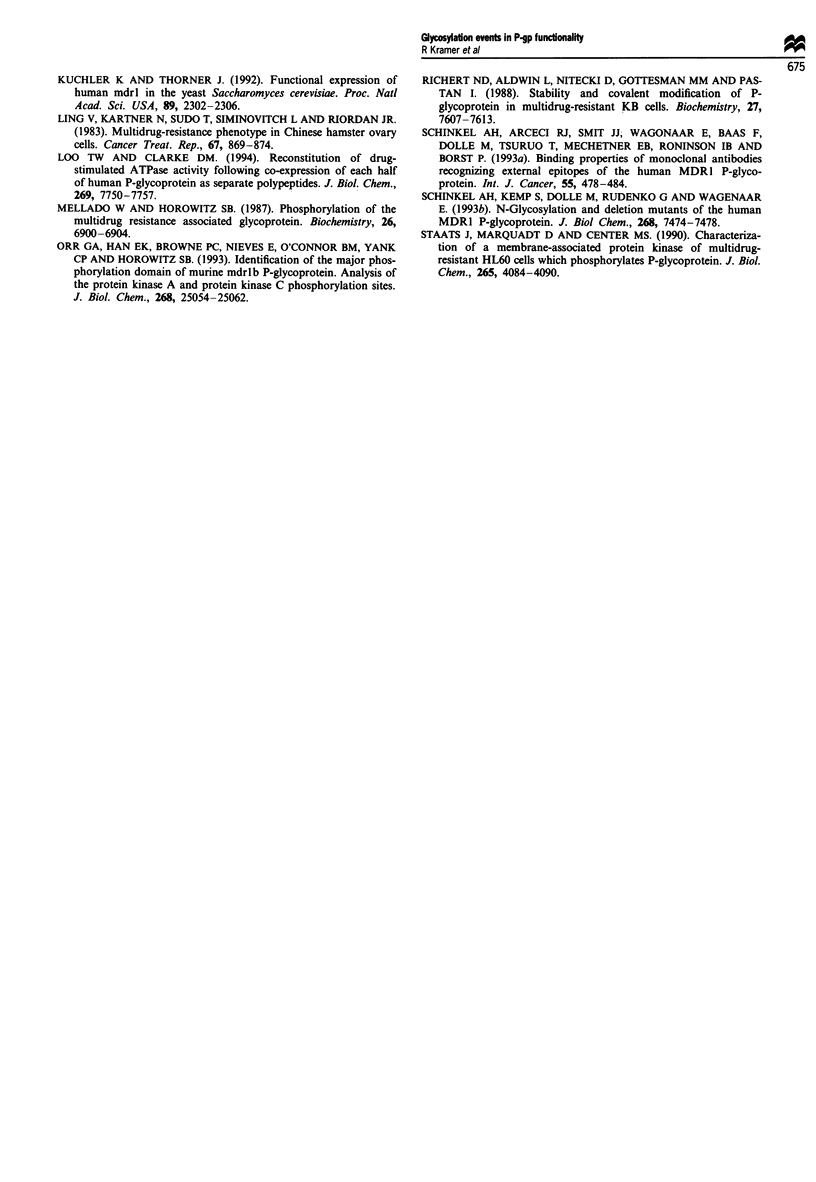# Inhibition of N-linked glycosylation of P-glycoprotein by tunicamycin results in a reduced multidrug resistance phenotype.

**DOI:** 10.1038/bjc.1995.133

**Published:** 1995-04

**Authors:** R. Kramer, T. K. Weber, R. Arceci, N. Ramchurren, W. V. Kastrinakis, G. Steele, I. C. Summerhayes

**Affiliations:** American Cyanamid Company, Medical Research Division, Pearl River, NY 10965, USA.

## Abstract

**Images:**


British Journal of Cancer (1995) 71, 670-675
© 1995 Stockton Press All rights reserved 0007-0920/95 $12.00

### Inhibition of *N*-linked glycosylation of P-glycoprotein by tunicamycin results in a reduced multidrug resistance phenotype

R Kramer[1], TK Weber[2], R Arceci[3], N Ramchurren[2], WV Kastrinakis[2], G Steele Jr[2] and IC Summerhayes[2]

[1]*American Cyanamid Company, Medical Research Division, Pearl River, NY 10965, USA;* [2]*New England Deaconess Hospital, Department of Surgery, Laboratory of Cancer Biology, 50 Binney Street, Boston, MA 02115, USA;* [3]*Dana-Farber Cancer Institute and Children's Hospital, Pediatric Hematology/Oncology, 44 Binney Street, Boston, MA 02115, USA.*

**Summary** Characterisation of altered glycosylation of P-glycoprotein (P-gp) found associated with the absence of a multidrug resistance (MDR) phenotype in cell lines prompted an investigation to assess the role of post-translational processing in establishing P-gp efflux pump functionality. The clone A cell line used in this study displays a strong MDR phenotype mediated by high constitutive levels of expression of P-gp. Incubation of clone A cells with tunicamycin for different periods resulted in a time-dependent increase in daunorubicin accumulation, reflecting a reduction in P-gp function. Parallel experiments conducted with verapamil resulted in no loss of P-gp functionality in clone A cells. Reduction in surface-associated P-gp following exposure to tunicamycin was established by FACS analysis, Western blot analysis and immunoprecipitation of surface-iodinated P-gp. In addition, immunoprecipitation of P-gp from $^{32}$P-orthophosphate-labelled cells demonstrated reduced phosphorylation of P-gp associated with tunicamycin exposure. From these studies we conclude that glycosylation of P-gp is required to establish the cellular MDR phenotype.

**Keywords:** P-glycoprotein; glycosylation; tunicamycin; MDR phenotype

Various steps in the biosynthesis of P-glycoprotein (P-gp) were characterised in a number of previous studies and demonstrated that P-gp is synthesised as a 140 kDa precursor, processed via *N*-linked glycosylation to a mature 170 kDa transmembrane glycoprotein (Greenberger *et al.*, 1988; Richert *et al.*, 1988; Loo and Clarke, 1994). A second step in the biosynthesis involves phosphorylation of the mature 170 kDa glycoprotein (Richert *et al.*, 1988), which is considered to be an essential event in establishing a fully functional efflux pump (Hamada *et al.*, 1987; Bates *et al.*, 1992; Kramer *et al.*, 1993*a*,*b*). Since the 140 kDa precursor molecule is not phosphorylated in the resting state (Kramer *et al.*, 1993*a*) and the mature 170 kDa protein is localised in the membrane, it is likely that phosphorylation is mediated by kinases acting at the plasma membrane (Staats *et al.*, 1990). Modulation of the phosphorylation and functional status of P-gp by the tumour promoter phorbol ester 4$\beta$-phorbol 12$\beta$ myristate 13$\alpha$-acetate suggests that protein kinase C (PKC) may be one kinase mediating this event (Hamada *et al.*, 1987; Chambers *et al.*, 1990, 1992). In addition, P-gp has been shown to be an *in vitro* substrate for protein kinase A (PKA) (Mellado and Horowitz, 1987), and both PKC and PKA phosphorylation domains have been identified (Orr *et al.*, 1993). Although the phosphorylation status of P-gp has been shown to be strongly correlated with the MDR phenotype in cell lines (Kramer *et al.*, 1993*a*), there is little direct evidence demonstrating a requirement for phosphorylation in establishing functionality. However, a recent study by Bates *et al.* (1992) has demonstrated an association between decreased P-gp phosphorylation and increased drug accumulation in a human colon cell line exposed to sodium butyrate. Moreover, antisense DNA directed against PKC[3] $\alpha$ has been shown to decrease the drug resistance of MCF-7/Adr cells (Ahmed and Glazer, 1993).

In contrast, glycosylation events associated with maturation of the 140 kDa precursor molecule are not considered important in establishing the functionality of the P-gp efflux pump (Chou and Kessel, 1981; Beck and Cirtain, 1982). Two lines of evidence suggest this to be the case. First, exposure of a drug-resistant cell line, CEM/VLB100, to pronase or tunicamycin does not diminish the activity of drug efflux in these cells (Beck and Cirtain, 1982). Second, colchicine-resistant hamster cell mutants displaying an altered carbohydrate moiety of P-gp retain a competent MDR phenotype in drug uptake assays (Ling *et al.*, 1983). Our previous studies, using a panel of human colon carcinoma cell lines, have identified two P-gp mutants in which altered processing of similar levels of the immature P-gp results in markedly different MDR phenotypes (Kramer *et al.*, 1993*a*). Underglycosylation of the immature 140 kDa P-gp in the Moser cell line results in an aberrant, mature 160 kDa protein which is phosphorylated, cell surface associated and capable of conferring the full MDR phenotype on these cells, confirming observations from earlier studies (Ling *et al.*, 1983). Cell line DLD-1 synthesises similar levels of immature 140 kDa precursor to Moser but displays little mature protein, minimal phosphorylation and greatly reduced cell-surface detectable P-gp. In contrast to the Moser cell line, DLD-1 is characterised by the absence of an MDR phenotype (Kramer *et al.*, 1993*a*). These findings raise questions as to the role of glycosylation in establishing a functional P-gp efflux pump. In this study we report the requirement for glycosylation of P-gp to establish a competent cellular MDR phenotype.

### Materials and methods

### Cell lines and drug exposure

Human colorectal carcinoma cell lines were maintained in culture in Dulbecco's modified Eagle medium (DMEM) supplemented with 5% fetal calf serum. Cell lines clone A and DLD-1 were provided by Dr D Dexter (DuPont De Nemours, Wilmington, DE, USA). Cell line MIP101 was established by Dr Niles (West Virginia, USA). The Moser cell line was kindly provided by Dr M Brattain (Medical College of Ohio, Toledo, OH, USA). The remaining cell lines were obtained from the American Type Culture Collection (ATCC). The P-gp status of the aforementioned cell lines has been previously established by this group (Kramer *et al.*,

Correspondence: IC Summerhayes
Received 13 June 1994; revised 4 November 1994; accepted 9 November 1994

1993*a*). Tunicamycin was added to complete medium at a final concentration of 2.5 μg $ml^{-1}$ (3 μM). Verapamil was used at a final concentration of 4 μg $ml^{-1}$ (10 μM).

*Immunoprecipitation of P-gp and epidermal growth factor receptor (EGFR)*

Subconfluent dishes of cell lines were washed twice in phosphate-buffered saline (PBS) and incubated for 1 h in methionine-free or phosphate-free DMEM followed by incubation in [$^{35}$S]methionine (150 μCi $ml^{-1}$) or [$^{32}$P]orthophosphate (150 μCi $ml^{-1}$) respectively. In pulse chase experiments cells were labelled for 10 min and chased in complete DMEM for different periods. In all other experiments labelling was for 3 h followed by immunoprecipitation of P-gp using the same protocol. Metabolically labelled cells were rinsed briefly in phosphate-buffered saline (PBS) and lysed in PBSTDS buffer (PBS pH 7.4, 1% Triton X-100, 0.5% sodium deoxycholate, 0.1% sodium dodecyl sulphate, 2 mM phenylmethylsulphonylfluoride, 10 U $ml^{-1}$ aprotinin). Lysis was carried out for 20 min at 4°C, followed by shearing of lysates through a 24 gauge needle. Lysates were clarified in a microfuge for 20 min at 4°C, supernatants were removed and 10 μl aliquots were taken for protein estimation using the bovine serum albumin (BSA) protein assay system (Pierce, Rockford, IL, USA). Before immunoprecipitation samples were standardised for protein concentration. Overnight incubation of lysates at 4°C with MDR-1 polyclonal antibody (Oncogene Science, Uniondale, NY, USA) was followed by a 90 min incubation with protein A–Sepharose beads. Immune complexes were washed three times in PBS then incubated in standard sample buffer for 20 min at room temperature. All samples were run on 7.5% polyacrylamide gels, dried and exposed for 1–3 days on X-ray film.

*Functional assays*

P-gp activity was determined using a daunorubicin accumulation assay (Kramer *et al.*, 1993*a*). Replicate suspensions of colon cells (2 × $10^6$ cells $ml^{-1}$) were incubated at 37°C for 90 min in medium containing daunorubicin (3 μM) in the presence or absence of verapamil or tunicamycin. In a separate experiment, cells were exposed to tunicamycin (2.5 μg $ml^{-1}$) or verapamil (4 μg $ml^{-1}$) for 24–36 h, washed twice in PBS and then assayed for daunorubicin accumulation, as described above. Cellular daunorubicin fluorescence was quantitated by flow cytometric analysis using Becton-Dickinson (Mountain View, CA, USA) FACSort and LYSYS II software.

*Flow cytometry*

Surface staining of cells for P-gp expression was accomplished using the 4E3 anti-Pg-p monoclonal antibody (MAb) (Arceci *et al.*, 1993). Adherent cells were collected in cold PBS by gentle scraping with a rubber policeman. Cells were washed twice in cold PBS and 1 × $10^6$ cells were resuspended in 100 μl of PBS containing a 1:1 dilution of human serum with PBS and incubated at 4°C for 30 min to block Fc receptors. Then, 2 ml of PBS was added to the cells, which were collected by centrifugation at 600 *g* for 3 min. Pelleted cells were resuspended in 100 μl of PBS containing 2% goat serum and 10 μg $ml^{-1}$ anti-P-gp MAb 4E3 or an IgG2a isotype-matched control antibody. This mixture was incubated for 30 min at 4°C. Cells were then washed twice with cold PBS and then resuspended in 100 μl of PBS containing 2% goat serum and fluorescein isothiocyanate (FITC)-labelled goat anti-mouse Ig $(Fab)_2$ fragment (TAGO) at a 1:30 dilution. Cells were incubated with the second antibody for 30 min at 4°C in the dark, then washed twice in cold PBS and fixed in 2% paraformaldehyde before analysis. The level of P-gp expression was determined using a Becton-Dickinson FACSscan II and LYSYS software application.

*$^{125}$I surface labelling of cells*

All procedures were carried out on ice. Cells to be labelled (5 × $10^6$) were suspended in 1 ml of PBS and then added to 0.5 mCi of neutralised Na$^{125}$I in 0.1 ml of PBS and lactoperoxidase (40 μg in water, 1 mg $ml^{-1}$). To start the reaction, 10 μl of hydrogen peroxide [30% (v/v) hydrogen peroxide in 10 ml of PBS] was added and mixed gently for 10 min. An additional 10 μl of hydrogen peroxide was added after this period followed by a final 10 μl of hydrogen peroxide 10 min later. Finally, 10 ml of PBS was added to the reaction mixture and the cells were pelleted by centrifugation. Cells were washed five times with 10 ml volumes of PBS, lysed in PBSTDS, clarified in a microfuge and precipitated overnight with MDR-1 antibody from 400 μg of total protein per sample (Kramer *et al.*, 1993*b*).

*Western blot analysis*

Crude membrane extracts were prepared from washed (PBS) pelleted cells harvested from dishes by scraping. Cells were exposed to hypotonic solution (10 mM Tris pH 7.2, 10 mM sodium chloride, 1.5 mM magnesium chloride, 1 mM dithiothreitol, 2 mM phenylmethylsulphonyl fluoride, 10 U $ml^{-1}$ aprotinin), vortexed and clarified in a microfuge (4000 *g*) for 5 min at 4°C. The supernatant was removed and the pellet resuspended in 1 ml of hypotonic solution, followed by incubation at 4°C for 15 min. The mixture was further disrupted in a Dounce homogeniser (60 strokes) and centrifuged at 7500 r.p.m. for 10 min at 4°C. The supernatants were removed and spun in an ultracentrifuge for 1 h (40 000 *g*) at 4°C. Pellets were resuspended in PBSTDS lysis buffer and protein concentrations were determined for each preparation. Total protein-standardised samples were immunoprecipitated using MDR-1 polyclonal antibody (Oncogene Science, Uniondale, NY, USA), resolved in 7.5% polyacrylamide gels and transferred overnight to nitrocellulose membrane. Blots were probed with MAb C219 (2 μg $ml^{-1}$) as recommended by the manufacturer (Centocor, Malvern, PA, USA) and developed using the ECL Western blotting detection system (Amersham, Aylesbury, UK).

### Results

*Effect of tunicamycin on P-gp synthesis*

To investigate the hypothesis that glycosylation of P-gp is required for the acquisition of the MDR phenotype in cells,

**Figure 1** Biosynthesis of P-gp in the human colon carcinoma cell line clone A. Pulse labelling of cells was performed in the presence of [$^{35}$S]methionine for a period of 10 min. Labelled cells were washed briefly and incubated in methionine-supplemented medium for different chase periods. Cells were then lysed and P-gp was immunoprecipitated as described in the Materials and methods section. Immunoprecipitated protein was separated on a 7.5% resolving gel, dried and exposed to X-ray film from between 1 and 3 days. The 140 kDa precursor (▶) and 170 kDa mature P-gp (▷) can be resolved. Numbered lanes represent different chase periods, lane 1, 0 h; lane 2, 2 h; lane 3, 4 h; lane 4, 6 h; lane 5, 8 h; lane 6, 10 h.

we repeated and extended the experiments of Beck and Cirtain (1982) using the MDR-competent colon carcinoma cell line clone A. Pulse-chase labelling experiments demonstrated P-gp synthesised as a 140 kDa precursor molecule in clone A, which is converted to the mature 170 kDa glycoprotein over a 4–6 h period (Figure 1), consistent with results reported by other groups (Richert *et al.*, 1988). Figure 2 shows immunoprecipitation of P-gp from clone A cells exposed to tunicamycin (2.5 $\mu$g ml$^{-1}$) demonstrating synthesis of precursor throughout continued exposure to the glycosylation inhibitor. Resolution of the 140 kDa precursor and 170 kDa mature P-gp was observed in the absence of tunicamycin (Figure 2, lane 1) and when label and drug were added together (Figure 2, lane 2). However, 6 h exposure to tunicamycin was sufficient to block completely glycosylation of newly synthesised P-gp (Figure 2, lane 3) resulting in an unglycosylated precursor molecule which migrates more rapidly than the previously characterised 140 kDa precursor (Figure 2, compare lower band in lanes 2 and 3). The migrational difference observed in repeated experiments is suggestive of some co-translation glycosylation events associated with the P-gp precursor product.

### *Functional assessment of tunicamycin on the MDR phenotype*

If glycosylation of P-gp is required for establishing the MDR phenotype, continuous maintenance of clone A cells in the presence of tunicamycin should compromise the functionality of the efflux pump. Figure 3 shows results from a study in which the zero time point demonstrates that the P-gp antagonist verapamil was able to increase the accumulation of daunorubicin by a factor of 4 when co-administered with daunorubicin, thus confirming the functional activity of P-gp in these cells. However, daunorubicin accumulation was not affected by co-administering tunicamycin over a range of doses in excess of those that block P-gp glycosylation, demonstrating that tunicamycin is not a MDR inhibitor in this system (Figure 3). These results contrast with those seen after exposing cells to verapamil or tunicamycin for 24 or 36 h (Figure 3) when cells were washed free of drug before conducting the daunorubicin accumulation assay. Under these conditions, verapamil had no effect on daunorubicin accumulation, whereas tunicamycin treatment resulted in a time-dependent increase in daunorubicin accumulation, reflecting a loss in P-gp function. These values were established over three separate experiments and represent a 5- to 7-fold reduction in pump efficacy compared with that recorded in untreated or in chronic verapamil-treated, clone A cells.

**Figure 2** Effect of tunicamycin on the biosynthesis of P-gp. Colon cell line, clone A, was incubated in the presence of tunicamycin (2.5 $\mu$g ml$^{-1}$) for different periods. Metabolic labelling of cells with [$^{35}$S]methionine was performed during the last 3 h of drug exposure. Cells were lysed and P-gp was immunoprecipitated as described in the Materials and methods section. The numbered lanes represent different periods of exposure to tunicamycin. Lane 1, 0 h; lane 2, 3 h; lane 3, 6 h; lane 4, 12 h; lane 5, 18 h; lane 6, 24 h; lane 7, 36 h.

### *Surface expression of P-gp*

Acquisition of the cellular MDR phenotype is likely to be dependent upon localisation of P-gp in the plasma membrane. To establish whether the compromised MDR phenotype observed in tunicamycin-treated clone A cells results from a reduction in surface-associated P-gp we used MAb 4E3 in FACS analysis on live cells. This antibody recognises an external epitope of human MDR-1 P-gp, independent of the glycosylation status of this molecule (Arceci *et al.*, 1993; Schinkel *et al.*, 1993*a*). Using this approach on clone A cells incubated in the presence of tunicamycin for 24 (Figure 4b), 36 (Figure 4c) and 48 (Figure 4d) h, revealed a reduction in surface-associated P-gp. Figure 4 shows approximately a 1.6-fold reduction in detectable cell-surface P-gp following 48 h exposure to tunicamycin.

**Figure 3** Functional assessment of P-gp activity in the presence of tunicamycin. P-gp activity was determined using a daunorubicin accumulation assay. Cells were maintained in the presence of verapamil (■, 4 $\mu$g ml$^{-1}$, 10 $\mu$M) or tunicamycin (▨, 2.5 $\mu$g ml$^{-1}$, 3 $\mu$M) for 0, 24 or 36 h. Cells were then washed free of drug and replicate suspensions ($2 \times 10^6$ cells ml$^{-1}$) were incubated at 37°C for 90 min in medium containing daunorubicin (3 $\mu$M). Untreated control values (□) at 24 and 36 h were within 10% of the time zero control values. Cellular daunorubicin fluorescence was quantitated by flow cytometric analysis.

**Figure 4** MDR-1 P-gp surface expression decreases with tunicamycin exposure. Clone A cells were stained with either the 4E3 MAb or an IgG2a isotype control following different time periods in the presence of tunicamycin as described in the Materials and methods section. (**a**) Clone A stained with IgG2a control (black histogram) *vs* 4E3 staining (white histogram). Black histograms in **b**, **c** and **d** represent the untreated clone A cells stained with 4E3 as a baseline comparison for 4E3 staining (white histograms) following different exposure times to tunicamycin: (**a**) 0 h; (**b**) 24 h; (**c**) 36 h; (**d**) 48 h.

*Membrane-associated expression of P-gp*

To characterise the level of expression and species, i.e. glycosylated or unglycosylated, of P-gp in the plasma membrane of tunicamycin-treated clone A cells, we prepared plasma membrane isolates of cells and probed protein-standardised immunoprecipitated lysates with MAb C219 in Western blot analysis. Figure 5 shows a representative experiment in which the 170 kDa mature P-gp is found in untreated clone A cells (Figure 5, lane 3) with a corresponding reduction in this glycoprotein in membrane preparations from clone A cells exposed to tunicamycin for different periods (Figure 5, lanes 4–6). In repeated experiments a second band, migrating with the unglycosylated precursor molecule, was faintly detected. To establish the possibility of the presence of membrane-associated precursor P-gp in tunicamycin-treated cells, we performed surface iodination of live cells followed by immunoprecipitation of P-gp from protein-standardised cell lysates. Figure 6 shows detection of only the mature (170 kDa) P-gp in untreated clone A cells (Figure 6, lane 3) with a reduction in the P-gp associated with exposure to tunicamycin (Figure 6, lanes 4 and 5), consistent with results of Western blot analysis. It is clear from these experiments that the unglycosylated P-gp (140 kDa) is iodinated in live cells and precipitated by the MDR-1 antibody in repeated assays.

**Figure 5** Western blot analysis of membrane-associated P-gp following exposure of clone A cells to tunicamycin. Crude membrane extracts were prepared from cell lines as outlined in the Materials and methods section. Total protein-standardised samples were immunoprecipitated using MDR-1 polyclonal antibody, resolved in 7.5% gels and transferred overnight to nitrocellulose membrane. Blots were probed with MAb C219 and developed using the ECL Western blot detection system. Lane 1, MIP-101, high-level expressor of membrane P-gp; lane 2, CCL 228, low-level expressor of membrane P-gp; lanes 3–6 represent membrane extracts from clone A cells exposed to tunicamycin for different periods. Lane 3, 0 h; lane 4, 12 h; lane 5, 24 h; lane 6, 36 h. Unmarked left lane shows 200 kDa molecular weight marker.

**Figure 6** Immunoprecipitation of P-gp from surface-iodinated colon carcinoma cells. Surface iodination of cells was performed as described in the Materials and methods section, followed by lysis and immunoprecipitation of P-gp using MDR-1 polyclonal antibody. Lane 1, MIP-101, high-level expressor of membrane P-gp; lane 2, CCL 228, low-level expressor of membrane P-gp; lane 3, clone A; lane 4, clone A exposed to tunicamycin for 12 h; lane 5, clone A exposed to tunicamycin for 24 h. Migration of the precursor (140 kDa) and mature (170 kDa) P-gp is denoted by arrows.

*Phosphorylation status of P-gp*

Our previous studies of colon carcinoma cell lines revealed phosphorylation associated solely with the mature 170 kDa P-gp, in which the phosphorylation level showed a strong correlation with the MDR phenotype of the cells (Kramer *et al*., 1993*a*). Immunoprecipitation of P-gp from cells labelled for 3 h with [$^{32}$P]orthophosphate revealed high levels of phosphorylation associated with the 170 kDa P-gp in untreated clone A cells (Figure 7, lane 1), consistent with our previous findings. In contrast, cells exposed to tunicamycin for extended periods displayed a significant reduction in phosphorylation associated with the mature P-gp (170 kDa) (Figure 7, lanes 2–4) with evidence of phosphorylation of the unglycosylated 140 kDa precursor molecule. The cell lines HT29 (Figure 7, lane 5) and MIP101 (Figure 7, lane 6) represent negative and positive P-gp controls respectively.

### Discussion

In this study we have evaluated the role of glycosylation of P-gp in establishing the cellular MDR phenotype. These studies were prompted by the identification of two P-gp mutants in a panel of human colon carcinoma cell lines which displayed altered processing of P-gp, resulting in contrasting MDR phenotypes (Kramer *et al*., 1993*a*). Both cell lines were established before chemotherapy and have not subsequently been exposed to known chemotherapeutic agents, hence representing constitutive expression of P-gp. The Moser cell line has previously been reported to synthesise a 140 kDa P-gp precursor molecule which displays an aberrant carbohydrate moiety (Kramer *et al*., 1993*a*) similar to that reported for P-gp in drug-selected Chinese hamster ovary cell lines (Ling *et al*., 1983). As with the hamster cell lines, the Moser line displays a competent MDR phenotype in which the altered mature P-gp is both membrane

**Figure 7** Phosphorylation status of P-gp in tunicamycin-exposed cells. Cells were incubated in the presence of [$^{32}$P]orthophosphate for 3 h followed by immunoprecipitation of P-gp as described in the Materials and methods section. Lanes 1–4 represent clone A cells incubated in the presence of tunicamycin (2.5 $\mu$g ml$^{-1}$) for different periods. Lane 1, 0 h; lane 2, 12 h; lane 3, 24 h; lane 4, 36 h, lane 5, HT29 negative control; lane 6, MIP101 positive control. Migration of the precursor (140 kDa) and mature (170 kDa) P-gp is denoted by arrows.

associated and phosphorylated, two features considered essential components of a completely functional cellular efflux pump. In contrast, DLD-1 synthesised similar levels of 140 kDa P-gp precursor to MDR-competent colon cell lines, e.g. clone A and Moser, and yet displayed little or no MDR phenotype in drug uptake assays (Kramer *et al.*, 1993*a*). Consistent with the idea that the 170 kDa P-gp is the phosphorylated membrane-associated species and is responsible for the MDR phenotype, DLD-1 has been shown to have greatly reduced surface-localised P-gp and minimal functionality in drug uptake assays (Kramer *et al.*, 1993*a*). These observations suggest that glycosylation of the precursor P-gp is important in establishing a competent MDR phenotype in these cells.

To address this issue in more detail, we have repeated and extended studies initially performed by Beck and Cirtain (1982), using the colon carcinoma cell line clone A, which has been shown to constitutively express P-gp and display a competent MDR phenotype (Kramer *et al.*, 1993*a*). In contrast to previous studies in which drug-resistant cells exposed to tunicamycin for 48 h maintained a competent MDR phenotype (Chou and Kessel, 1981; Beck and Cirtain, 1982), we found increased drug retention in clone A cells exposed to tunicamycin suggestive of a role for glycosylation in establishing the P-gp-mediated MDR phenotype. Following exposure of clone A cells to tunicamycin (2.5 $\mu g\ ml^{-1}$) for 36 h, retention of daunorubicin was 5- to 7-fold greater than that recorded in clone A cells exposed to verapamil (4 $\mu g\ ml^{-1}$) for the same period or in untreated clone A cells. Interestingly, FACS analysis of tunicamycin-exposed clone A cells, using MAb 4E3, revealed only a 1.6-fold reduction in cell-surface-associated P-gp following 48 h of tunicamycin exposure, although surface iodination and Western blot analysis suggest more significant reductions in membrane-associated P-gp. With the demonstrated effective inhibition of glycosylation by tunicamycin on newly synthesised P-gp, why is the MDR phenotype not completely abrogated? Given that the viability of cells exposed to tunicamycin drops rapidly after 48 h and that the half-life of P-gp is between 48 and 72 h (Richert *et al.*, 1988), it is clear that a significant proportion of presynthesised P-gp will remain throughout the time course of experiments in this study. Hence, with this approach one would not expect to abolish the P-gp-mediated MDR phenotype within the time frame of these studies. Given this limitation, one explanation for the discrepancy between our results and those of Beck and Cirtain (1982) could relate to the level of expression of P-gp in the cell lines used. The cell line CEM/VLB100 was selected for resistance to vinblastine and expresses high levels of P-gp. Although we have no directly comparable data with clone A, it is likely that the level of P-gp expression in CEM/VLB100 is greater than recorded in clone A, as is the case with other drug-selected cell lines (Arceci *et al.*, 1993). In such circumstances the elevated levels of P-gp together with the extended half-life of the protein combine to maintain sufficient P-gp in the presence of tunicamycin to cope with drug efflux at the concentrations used in drug uptake assays. Hence, only cell lines expressing lower levels of P-gp would exhibit a compromised MDR phenotype in the prsence of tunicamycin. However, should tunicamycin exposure result in premature degradation of newly synthesised P-gp, then reduced overall levels of P-gp could account for the loss of MDR phenotype.

Previous studies involving the characterisation of P-gp glycosylation mutants demonstrated that altered glycosylation of P-gp can occur without compromising the MDR phenotype (Ling *et al.*, 1983). We have found this to be true in the Moser cell line, in which the altered carbohydrate moiety of the mature P-gp does not prevent membrane localisation and phosphorylation of P-gp resulting in a full MDR phenotype. Whether the unglycosylated 140 kDa P-gp precursor can maintain the MDR phenotype *in vivo* is unclear, although it has been shown that a functional pump can be established in yeast in the absence of glycosylation (Kuchler and Thorner, 1992). Iodination studies of clone A cells exposed to tunicamycin suggest that some unglycosylated P-gp is localised at the cell surface under these conditions. Since phosphorylation is considered to be an integral component in the functionality of P-gp, it is interesting to note that phosphorylation of a 140 kDa protein is also detected in tunicamycin-exposed clone A cells. We have never observed this in any case of constitutive expression of P-gp in an extended series of cell lines studied (Kramer *et al.*, 1993*a*). Although it is evident that tunicamycin, resulting in the inhibition of glycosylation, can affect the efficacy of the P-gp-mediated efflux pump, it is likely that this is as a consequence of a combination of factors. These include loss of cell-surface-associated P-gp and reduced phosphorylation of persisting P-gp in cells. Although unglycosylated P-gp may prove functional in extracellular assays, the perturbation of translocation to the membrane will compromise its efficacy as a cellular detoxification pathway. These results are consistent with a recent report involving mutation of the conserved *N*-glycosylation sites of the human P-gp, the findings of which suggest that glycosylation contributes to proper routing or stability of P-gp (Schinkel *et al.*, 1993*b*). In this way glycosylation does not contribute to functional aspects of the P-gp pump *per se* but is required in establishing competent cellular MDR phenotypes.

**Acknowledgements**
We would like to thank Carol Ann Hannan for manuscript preparation. This work was supported by National Institutes of Health Grants CA42944 and CA44704.

**References**

AHMAD S AND GLAZER RI. (1993). Expression of the antisense cDNA for protein kinase C alpha attenuates resistance in doxorubicin-resistant MCF-7 breast carcinoma cells. *Mol. Pharmacol.*, **43**, 858–862.

ARCECI RJ, STIEGLITZ K, BRAS J, SCHINKEL A, BAAS F AND CROOP J. (1993). Monoclonal antibody to an external epitope of the human mdr1 P-glycoprotein. *Cancer Res.*, **53**, 310–317.

BATES SE, CURRIER SJ, ALVAREZ M AND FOJO AY. (1992). Modulation of P-glycoprotein phosphorylation and drug transport by sodium butyrate. *Biochemistry*, **31**, 6366–6372.

BECK WT AND CIRTAIN MC. (1982). Continued expression of vinca alkaloid resistance by CCRF-CEM cells after treatment with tunicamycin or pronase. *Cancer Res.*, **42**, 184–189.

CHAMBERS TC, MCAVOY EM, JACOBS JW AND EILON G. (1990). Protein kinase C phosphorylates P-glycoprotein in multidrug-resistant human KB carcinoma cells. *J. Biol. Chem.*, **265**, 7679–7686.

CHAMBERS TC, ZHENG B AND KUO JF. (1992). Regulation by phorbol ester and protein kinase C inhibitors and by a protein phosphatase inhibitor (okadaic acid), of P-glycoprotein phosphorylation and relationship to drug accumulation in multidrug-resistant human KB cells. *Mol. Pharmacol.*, **41**, 1008–1015.

CHOU T-H AND KESSEL D. (1981). Effects of tunicamycin on anthracycline resistance in P388 murine leukemia cells. *Biochem. Pharmacol.*, **30**, 3134–3136.

GREENBERGER LM, WILLIAMS SS, GEORGES E, LING V AND HOROWITZ SB. (1988). Electrophoretic analysis of P-glycoproteins produced by mouse J774.2 and Chinese hamster ovary multidrug-resistant cells. *J. Natl Cancer Inst.*, **80**, 506–510.

HAMADA H, HAGIWARA K-I, NAKAJIMA T AND TSURUO T. (1987). Phosphorylation of the Mr 170,000 to 180,000 glycoprotein specific to multidrug-resistant tumor cells: effects of verapamil, trifluoperazine and phorbol esters. *Cancer Res.*, **47**, 2860–2865.

KRAMER R, WEBER TK, MORSE B, ARCECI R, STANIUNAS R, STEELE Jr G AND SUMMERHAYES IC. (1993*a*). Constitutive expression of multidrug resistance in human colorectal tumors and cell lines. *Br. J. Cancer*, **67**, 959–968.

KRAMER R, WEBER TK, ARCECI R, MORSE B, SIMPSON H, STEELE Jr GD AND SUMMERHAYES IC. (1993*b*). Modulation of MDR-1 expression by a H-ras oncogene in a human colon carcinoma cell line. *Int. J. Cancer*, **54**, 275–281.

KUCHLER K AND THORNER J. (1992). Functional expression of human mdr1 in the yeast *Saccharomyces cerevisiae*. *Proc. Natl Acad. Sci. USA*, **89**, 2302–2306.

LING V, KARTNER N, SUDO T, SIMINOVITCH L AND RIORDAN JR. (1983). Multidrug-resistance phenotype in Chinese hamster ovary cells. *Cancer Treat. Rep.*, **67**, 869–874.

LOO TW AND CLARKE DM. (1994). Reconstitution of drug-stimulated ATPase activity following co-expression of each half of human P-glycoprotein as separate polypeptides. *J. Biol. Chem.*, **269**, 7750–7757.

MELLADO W AND HOROWITZ SB. (1987). Phosphorylation of the multidrug resistance associated glycoprotein. *Biochemistry*, **26**, 6900–6904.

ORR GA, HAN EK, BROWNE PC, NIEVES E, O'CONNOR BM, YANK CP AND HOROWITZ SB. (1993). Identification of the major phosphorylation domain of murine mdr1b P-glycoprotein. Analysis of the protein kinase A and protein kinase C phosphorylation sites. *J. Biol. Chem.*, **268**, 25054–25062.

RICHERT ND, ALDWIN L, NITECKI D, GOTTESMAN MM AND PASTAN I. (1988). Stability and covalent modification of P-glycoprotein in multidrug-resistant KB cells. *Biochemistry*, **27**, 7607–7613.

SCHINKEL AH, ARCECI RJ, SMIT JJ, WAGONAAR E, BAAS F, DOLLE M, TSURUO T, MECHETNER EB, RONINSON IB AND BORST P. (1993*a*). Binding properties of monoclonal antibodies recognizing external epitopes of the human MDR1 P-glycoprotein. *Int. J. Cancer*, **55**, 478–484.

SCHINKEL AH, KEMP S, DOLLE M, RUDENKO G AND WAGENAAR E. (1993*b*). N-Glycosylation and deletion mutants of the human MDR1 P-glycoprotein. *J. Biol Chem.*, **268**, 7474–7478.

STAATS J, MARQUADT D AND CENTER MS. (1990). Characterization of a membrane-associated protein kinase of multidrug-resistant HL60 cells which phosphorylates P-glycoprotein. *J. Biol. Chem.*, **265**, 4084–4090.